# Cardiovascular inhalation for targeted drug delivery in cardiac disease

**DOI:** 10.1007/s10741-025-10527-w

**Published:** 2025-05-23

**Authors:** Alessio Alogna, Francesco Paolo Lo Muzio, Daniele Catalucci

**Affiliations:** 1 Deutsches Herzzentrum Der Charité, Department of Cardiology, Angiology and Intensive Care Medicine, Campus Virchow-Klinikum, Augustenburgerplatz 1, 13353 Berlin, Germany; 2 DZHK (German Centre for Cardiovascular Research), Partner Site, 10785 Berlin, Germany; 3 Institute of Genetic and Biomedical Research (IRGB), National Research Council of Italy, Milan Unit, 20138 Milan, Italy; 4 IRCCS Humanitas Research Hospital, 20089 Rozzano, Milan Italy

**Keywords:** Cardiovascular inhalation, Nanoparticles, Cardiac targeting, Peptides, Biologics, Heart failure

## Abstract

Recombinant proteins, cell, and gene therapies are collectively defined as biological drugs or biologics. These therapies have transformed the lives of millions of patients over the past decades, with the number of FDA-approved biologics increasing exponentially in recent years. However, out of approximately 700 biological therapies approved by the FDA in the last 20 years, less than 1% are indicated for cardiac pathologies. The application of biologics in cardiovascular disease has faced significant challenges, including short plasma half-life, the multifactorial complexity of cardiac disease, and the lack of efficient, non-invasive, and patient-friendly drug-delivery routes. This translational gap is particularly pressing given the immense socioeconomic burden of cardiovascular disease, which remains the leading cause of death globally and accounts for billions in annual healthcare costs and lost productivity. Inhalation-based drug delivery has recently emerged as a promising strategy for treating cardiovascular disease, with several proof-of-concept studies demonstrating its potential in heart failure, the most prevalent cardiac condition. This narrative review summarizes the latest experimental evidence in the novel field of *Cardiovascular Inhalation*, i.e., the lung-to-heart route for biologics. We discuss translational challenges, preclinical evidence, and future perspectives for bringing this innovative approach to clinical practice.

## Introduction

We are currently experiencing the advent of biologics in cardiovascular medicine. Recent scientific research has focused on peptides, i.e., short chain of amino acids, as well as nucleic acids, particularly microRNAs (miRNAs), as promising therapeutic agents for a multitude of diseases. Accordingly, growing evidence shows that miRNAs play an important role in the onset and progression of cardiovascular disease [1, 2]. Although several miRNAs have emerged as promising therapeutic candidates in cardiovascular medicine, the majority of clinical trials are focusing on circulating miRNAs as diagnostic or prognostic biomarkers. The use of miRNA antisense as therapeutic for heart disease is limited to only one active clinical trial (NCT05350969). The reason for that is related to the knowledge of the multi-target effect of miRNAs potentially inducing unwanted off-target side effects such as thrombocytopenia or severe immune response [2]. Despite their promising potential, delivering these molecules preferentially into cardiomyocytes remains a significant challenge, further complicated by their instability and rapid degradation in the body. While chemical modifications can enhance stability, as demonstrated with miRNA antisense, achieving the right balance between efficacy and safety remains challenging for synthetic miRNA mimics. Currently, cardiac delivery of biologics in large mammals requires invasive approaches and their enrichment at the heart strongly varies with the route of administration, e.g., intramyocardial injection, intracoronary infusion, or carrier used, e.g., nanoparticles or viral vectors [3]. For instance, adeno-associated viruses (AAVs), typical harmless viruses used as drug-carriers, have been adopted but are difficult to direct to target individual tissues, and they simply do not work in up to 40% of people because they have already been exposed to them in life and they produce antibodies against them [4]. In addition, prolonged, uncontrolled, and persistent expression of delivered therapeutics can be harmful and potentially lethal [5]. Genotoxicity may arise from rare viral vector integration into the genome [6]. Intramyocardial injection overcomes blood interaction and the endothelial barrier, while obviously needle injury is an important concern. Moreover, the volume injected can be a determinant of myocardial leakage or myocardial embolization and plugging.

In summary, the lack of an efficient delivery system capable to allow preferential cardiac uptake, controlled drug release, low toxic side effects, and low-dose administration remains a major issue for the translatability of novel therapeutic compounds.

### Inhalation for systemic delivery of biologics

**Fig. 1:**
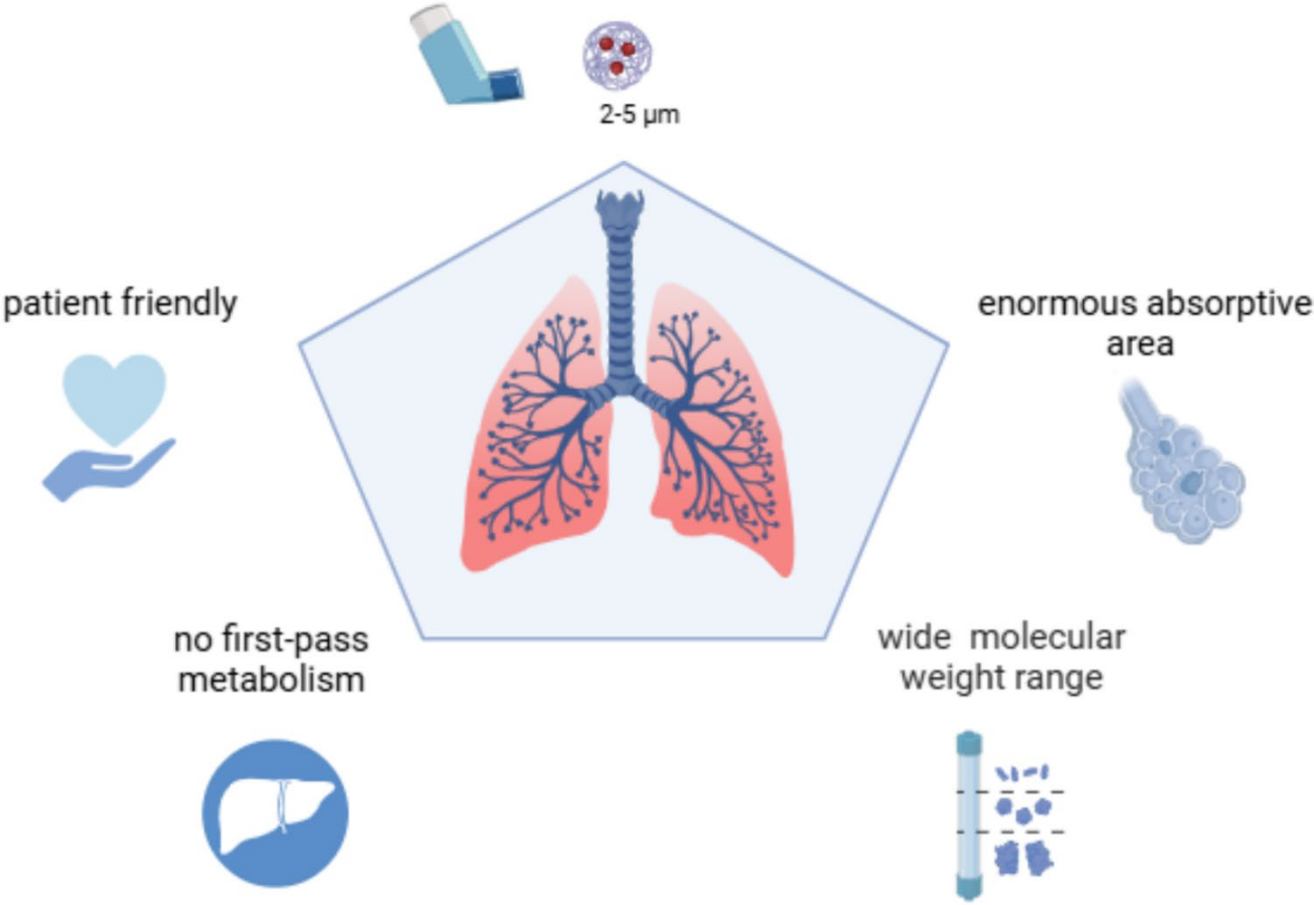
Major advantages of pulmonary drug delivery for biologics

Inhaled drugs are well established for the treatment of lung-disease, in particular asthma and chronic obstructive pulmonary disease [7]. The idea of applying the inhalation route for treating diseases beyond the lung has been thoroughly explored as it offers several advantages, as summarized in Fig. 1: (i) It is patient friendly, while (ii) directly delivering the therapeutic compound to the systemic circulation, therefore bypassing the first-pass metabolism in the liver [7]; (iii) it is suitable for a wide range of substances from small molecules to very large proteins [8], given the (iv) enormous lung surface with a easily permeable membrane such as the alveolo-capillary membrane. However, previous attempts to leverage this administration route have mostly been disappointing. The long-term use of inhalable insulin for diabetic patients has been documented to induce wheezing and bronchoconstriction and, potentially, gradual loss of lung function [9]. Inhalable levodopa showed promising results in different clinical trials for Parkinson’s disease, but again the most common adverse event was bronchospasm, which was found in approximately15% of patients in the SPAN-PD trial [10].

With the growing relevance of peptides and miRNAs in the pharmaceutical industry, there is a new wave of significant research and development interest in exploring less-invasive or non-invasive methods for the systemic delivery of biologics, such as inhalation [11]. The development of biologics for inhalation presents several challenges, including proper deposition and distribution in the deep lungs, and the complex permeation through the pulmonary surfactant and the alveolocapillary membrane, all while avoiding resident macrophages and ubiquitarian enzymes [11]. Several inhalable formulation and devices have therefore been developed in the past decades with the aim of enhancing pulmonary drug deposition, and dry powder (dp) inhalers have emerged as the preferred method for delivering inhalable biologics, because of the stability of dp-formulated biologics for storage [12]. To enable the pulmonary delivery, formulating effective drug powders is essential. Most inhalable drug powders consist of particulate carriers, with various synthesis methods—such as spray drying, milling, and freeze-drying—allowing for optimization of formulation parameters. Early research established that characteristics such as particle density, size (preferentially in the range of 2–5 µm), porosity, and charge significantly influence lung deposition and drug absorption, boosting the clinical translation of several formulations for clinical use [11].

Overall, the development of inhalation formulations for biologics targeting organs other than lungs requires a substantial paradigm shift for the accomplishment of a complete preclinical package in terms of pharmacokinetics, pharmacodynamics, and toxicity studies. The most obvious example is related to the peak plasmatic concentration of an inhaled compound, e.g., COPD or asthma spray, which is rather seen as a drug spill-over to be avoided. Indeed, these drugs should exert their effect only locally, i.e., in the lungs. On the contrary, inhaled drugs for systemic administration are supposed to exert their on-target effect shortly after hitting the systemic circulation, avoiding the trap of type II alveolar epithelial cells, i.e., macrophages, in order to minimize any deposition and related inflammatory reactions in the lungs. Furthermore, toxicity studies are of paramount importance in order to detect early signs of airways hyperreactivity related to a novel formulation, especially after long-term exposure to daily inhalation. Challenges related to delivering biologics via the inhaled route are summarized in an excellent overview published in 2021 as a result of a cross-industry working-group survey [13].

Building on these challenges, understanding the underlying chemical principles of inhaled biologics and their formulation strategies is essential to optimizing therapeutic outcomes. The development of inhaled biologics requires careful consideration of their chemical and physicochemical properties, including molecular weight, solubility, and stability during aerosolization. Synthetic peptides and nucleic acids, for example, often require encapsulation in nanocarriers to protect them from enzymatic degradation and facilitate pulmonary absorption, as these biomolecules are typically unstable in the extracellular lung environment [11, 12]. Small molecules, depending on their physicochemical traits, can be formulated as dry powders or solutions, with excipients enhancing dispersibility and lung deposition [12]. Both conventional drugs and experimental biologics have been evaluated for inhalation delivery; while conventional drugs may rely on straightforward formulations, novel biomaterials often demand advanced engineering to ensure bioavailability and tissue targeting [13].

Dosing for inhaled biologics must be carefully optimized to ensure sufficient systemic exposure while minimizing local pulmonary side effects [14]. Unlike oral or intravenous routes, inhalation allows for lower doses due to rapid alveolar absorption and avoidance of hepatic first-pass metabolism. However, factors such as particle size, deposition efficiency, and mucociliary clearance can significantly influence bioavailability and must be further optimized in patients with pathological lung conditions such as pulmonary congestion. Tailoring dose delivery through patient-adapted inhalation devices and consistent dry powder performance will be crucial for maintaining therapeutic exposure across patient populations and should be accounted for during dose selection and formulation development.

*Cardiovascular Inhalation* as a novel therapeutic paradigm.

Among the potential organs to target via inhalation, the heart has emerged as a promising candidate due to the growing number of novel therapeutic biologics for cardiac diseases [15] and the lack of safe, effective delivery methods for targeted administration [3]. Here, we introduce the term *cardiovascular inhalation* to describe the novel approach of using inhaled therapeutics to target the heart. Although still in its early stages, this concept is attracting significant attention for its potential in various therapeutic applications [16].

To fully exploit the potential of *cardiovascular inhalation*, careful selection of appropriate drug candidates is critical. The selection of therapeutic candidates for cardiovascular inhalation therapy requires a strategic evaluation of several key parameters. Molecules must exhibit adequate physicochemical stability during aerosolization and resistance to enzymatic degradation within the lung environment. Furthermore, they should demonstrate sufficient permeability across the alveolar-capillary barrier and favourable pharmacokinetics for systemic or targeted cardiac uptake. Candidates such as short peptides, RNA-based molecules (e.g., miRNA mimics), and small hydrophilic drugs are particularly suitable due to their size, modifiability, and established preclinical efficacy. In addition, drugs with well-characterized mechanisms of action in cardiac pathophysiology provide a rational basis for translational development via inhalation. Ultimately, the goal is to identify compounds that balance lung safety, systemic bioavailability, and cardiac selectivity, either through inherent properties or via nanocarrier-based targeting strategies [11, 16].

In the mammalian circulatory system, blood from the lungs flows directly to the heart via the pulmonary vein after gas exchange. This direct pathway presents a unique opportunity to deliver peptides or miRNAs via inhalation, provided that an efficient carrier facilitates alveolar transcrossing, protects the biologics from plasmatic degradation, and ensures targeted cellular internalization. This process, which we have termed the lung-to-heart administration route [17], leverages the pulmonary circulation for direct cardiac access. However, a primary technical challenge lies in achieving an optimal formulation size: while particles in the 3–5-µm range are needed to reach the deep lung, avoiding direct exhalation, further reduction to < 2 µm is necessary to cross the alveolar-capillary barrier and ensure effective delivery to the heart [13]. An ideal dp formulation consists then of microparticles embedding the drug-loaded nanoparticles (NanoInMicro technology, NIM).

#### Nanocarriers are needed for cardiovascular inhalation

**Fig. 2:**
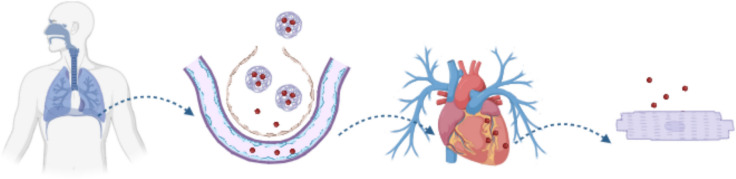
Lung-to-heart journey: from left to right: (i) oral inhalation, (ii) microparticles dissolve within the surfactant in the alveoli, (iii) translocation in the systemic circulation of the loaded nanoparticles, and (iv) the drug is entering the cardiomyocytes via endocytosis and released intracellularly via an effective endosomal escape

Figure 2 summarizes the lung-to-heart journey. After reaching the deep-lung, the dp microparticles need to dissolve within the surfactant in the alveoli and release the nanoparticles, which translocate into the bloodstream [17]. Nanoparticles (NPs) provide a strategy for an efficient, controlled, and safe drug-delivery. Indeed, NPs can potentially bind and deliver a large plethora of agents including peptides and miRNAs, protecting them from degradation and controlling drug release. Most inhaled nanoparticles tend to localize in the alveolar region, where they release their therapeutic payload gradually. Current research in nanoparticle-based inhalation therapies focuses on reducing adverse effects, improving treatment efficacy, and refining drug delivery specifically to lung tissues. Advanced nanoparticle platforms for inhalation-based delivery have been comprehensively characterized in a recent review [18].

Overall, three major classes of inhalable NPs have emerged in recent years, including polymeric nanoparticles (PNPs), lipid-based nanoparticles (LNPs), and inorganic nanoparticles (INPs). PNPs, such as those composed of chitosan, poly(lactic-co-glycolic acid) (PLGA), or polyethyleneimine (PEI), offer controlled drug release and protection from enzymatic degradation while exhibiting favorable physicochemical properties for inhalation. Chitosan-based nanoparticles, for instance, demonstrate mucoadhesive properties that enhance pulmonary retention and absorption due to their interaction with mucus glycoproteins and positive surface charge, which promotes epithelial uptake [19]. PLGA-based formulations, on the other hand, have been optimized for inhaled therapy via tailoring the porosity [20] as well as combining the high lung deposition advantage of microparticles with a nanoparticle in microparticle delivery system (NIM), which can quickly release free nanoparticles after lung delivery, so as to effectively overcome the lung clearance barrier and achieve good systemic therapeutic effect [21]. Their advantages for inhalation include biodegradability, controlled release, and ability to encapsulate both hydrophilic and hydrophobic drugs.

LNPs, including solid lipid nanoparticles (SLNs) and nanovesicles such as liposomes, have been widely investigated due to their ability to efficiently encapsulate bioactive molecules and facilitate cellular uptake [8]. Notably, exosome-based LNPs have gained attention in cardiovascular research for their role in delivering miRNAs and proteins involved in cardioprotection and tissue regeneration [22]. Exosomes are nanoscale extracellular vesicles of endosomal origin, secreted by progenitor cells, and several studies have demonstrated the dose-dependent efficacy of exosome therapy in improving cardiac function [23, 24]. Building upon this evidence, fibroblast-derived cardioprotective exosomes have recently been shown to ameliorate cardiac remodeling and improve function in preclinical heart failure, offering a scalable, noninvasive therapeutic approach [25]. In contrast, NPs composed of synthetic polymers and micelles often suffer from scarce biocompatibility, low encapsulation efficacy, and slow biodegradability [26]. For cardiac applications, it is well established that nanoparticle systems must meet specific physicochemical criteria—namely, colloidal stability, controlled size and shape, and a negatively charged surface—to avoid interference with cardiomyocyte function [27, 28].

Among INPs, negatively charged calcium phosphate nanoparticles (CaP-NPs) are characterized by high biocompatibility and pH-sensitive stability, which facilitates the release of their load in biological acidic environments (i.e., endosomes or lysosomes) [28]. The CaP-NPs have a platelet-like shape with a solid diameter of 20–50 nm and a hydrodynamic mean diameter < 200 nm, thus being in the perfect size for the inhalation route. The mechanism of cell internalization follows the conventional physiological process of clathrin- and dynamin-mediated endocytosis, thus without inducing toxicity or apoptosis. Moreover, the complete dissolution in Ca^2+^ and ${\text{PO}}_{4}^{3-}$, the main constituents of CaP-NPs, prevents unwanted NP accumulation in cells and tissues. As shown by Di Mauro et al., CaP-NPs do not interfere with functional properties of the cardiomyocytes both in vitro and in vivo [28]. Following on this development, Miragoli et al. demonstrated the feasibility of using CaP-NPs and inhalation as an approach for delivering therapeutic peptides to the heart in vivo in mouse, rats, and pigs [29, 30]. Recently, Modica et al. verified the therapeutic application of these CaP-NPs as carriers of the miR133a mimic in a pressure-overload heart failure (HF) mouse model [31]. Finally, Quarta et al. produced a microparticulate dp product out of the peptide-loaded CaP-NPs to be inhaled from a commercially available inhalation device (e.g., DPI), thus improving their stability and making the dose control more predictable [32]. In their design of experiment, the formulation was achieved by spray-drying nanoparticle dispersions in an aqueous solution of mannitol, a common polyalcohol already used in the clinical treatment of cystic fibrosis patients. The resulting formulations were defined as the lung-to-heart nano-in-micro-technology (LungToHeartNIM).

## Preclinical evidence for cardiovascular inhalation in heart failure

Several drugs have been developed and tested in large, randomized clinical trials, leading to the establishment of the so-called four pillars of HF with reduced ejection fraction (HFrEF) therapy. Those therapies, including beta-blockers, ACE-inhibitors/AR-blockers, mineralocorticoid receptor antagonists, and SGLT-2 inhibitors, primarily alleviate symptoms and slow disease progression. However, the need for novel treatments addressing the underlying molecular dysfunctions, i.e., disease-modifying drugs, remains particularly high [33].

The evidence of inhaled therapies for HF, both reduced and preserved ejection fraction (HFpEF), are summarized in Table 1. While the most of the inhalation approaches have been testing synthetic drugs, we will focus on two recent examples of successful inhalation of biologics in HFrEF [17] and HFpEF [34], respectively.

**Table 1:** List of preclinical studies investigating inhaled therapies for cardiovascular disease treatment

Study (by year)	Therapeutic agent	Animal model	Delivery method	Outcome	
Hunter C. et al., Nature Medicine, 2004	Sodium nitrite	Newborn lambs with pulmonary hypertension	Nebulization	Improved pulmonary artery pressures	
Aguero J. et al., Journal of the American College of Cardiology, 2016	AAV1.SERCA2a	Chronic post-capillary pulmonary hypertension in Yorkshire swine	Nebulization	Prevented disease progression as evaluated by mean pulmonary artery pressure, vascular resistance, and limited vascular remodeling quantified by histology	
Miragoli et al., Science Translational Medicine, 2018	Therapeutic peptide-loaded calcium phosphate nanoparticles	Mice with diabetic cardiomyopathy and healthy swine	Nebulization	Restored cardiac function in diabetic cardiomyopathy	
Watanabe S. et al., Pulmonary Circulation, 2018	AAV1.SERCA2a	Yukatan miniature swine model of chronic pulmonary hypertension	Nebulization	homogenous distribution of vectors, and lower pulmonary vascular resistance	
Alogna et al., Journal of the American College of Cardiology, 2024	Therapeutic peptide (LungToHeartNiM)	Pigs with non-ischemic heart failure	DP Inhalation	Improved left ventricular contractility, well-tolerated in large animal model	
Li J. et al., Circulation, 2024	Inhaled stem cell exosomes	Mice and swine post-myocardial infarction	Nebulization	Ameliorated cardiac repair, improved cardiac function and reduced fibrosis	
Weng et al., Nature Communications, 2024	Novel peptide-loaded nanoparticles	Mice with transverse aortic constriction (TAC)	Nebulization	Reduced hypertrophy in the left ventricle, improved cardiac function, decreased fibrosis and adverse remodeling	

The pathophysiology of HFrEF involves multiple altered signaling pathways, which triggers the phenotypic changes in size, shape, and function of the heart resulting in a decreased pumping capacity. At subcellular level, a key player of the pathology is the L-type Ca^2^⁺ channel (LTCC), which is critical for the cardiac excitation–contraction coupling process. In HFrEF occurs functional alterations in the plasma membrane density, subcellular localization, and gating properties of the LTCCs leading to a defective Ca^2+^ machinery [35–37]. Therefore, investigation on positive Ca^2+^ modulators might be pivotal in addressing one of the main mechanisms of the impaired contractility observed in HFrEF. However, pharmacological approaches aiming to enhance inotropism in HFrEF have been found to promote arrhythmogenesis and diastolic dysfunction, thereby limiting their use in the clinic [38].

In the past years, we showed that a therapeutic candidate with great potential is the cell-penetrating mimetic peptide (R7 W-MP), which has the capability to modulate the Ca_v_β2 cytosolic subunit of the LTCC [39]. Of notice, without the cell-penetrating sequence, the MP loses the capability to enter the cardiac cell, an issue that can be solved by association in CaP-NPs protecting the peptide from degradation. To fully exploit the inhalation route, we have developed the abovementioned LungToHeartNIM by embedding CaP-MP in inhalable dry powder microparticles (dpCaP-MP) [32]. The novel drug and inhalation approach was tested in a clinically relevant pig model of chronic non-ischemic HFrEF [17], i.e., tachypacing-induced dilative cardiomyopathy. A daily inhalation of the dpCaP-MP (MP 60 μg/kg) for 2 weeks induced a restoration of cardiac function with LVEF above 50%, improved lung congestion, HF symptoms, and NT-proBNP. Finally, proteomic analysis showed that dpCaP-MP inhalation triggers a reverse modelling cascade in which the protein expression profile reverts to that of healthy animals. At the single cardiomyocyte level, immunofluorescence analysis for LTCC and ryanodine receptor (RyR2) demonstrated that the therapeutic effect of the novel drug involves realignment of the calcium channel to the RyR2, restoring the subcellular colocalization essential for correct excitation–contraction coupling. No therapy-related and drug-related adverse effects, nor alterations of calcium and phosphate levels in the blood, were detected during the trial [17]. The LungToHeartNIM peptide platform represents a groundbreaking approach in the treatment of HFrEF providing a non-viral, safe, versatile, and scalable method for delivering biologics to the heart, paving the way for targeting various cardiac and non-cardiac diseases.

Recent research in HFpEF has leveraged the same CaP nanotechnology, slightly modified for further cardiomyocyte enrichment enhancement, for delivering TP-10, a selective inhibitor of phosphodiesterase 10 A [34]. This approach aims to improve drug delivery efficiency directly to cardiac tissues, addressing the limitations of traditional systemic therapies that often suffer from poor targeting and off-target effects. The inhalation of TP-10 led to improved cardiac function and reduced remodeling in a pressure-overload murine model, already with a low dosage regimen (2.5 mg/kg every 2 days) that minimizes lung injury risks. The therapeutic effects of this inhalable system are mediated through critical signaling pathways, specifically cAMP/AMPK and cGMP/PKG, which are crucial in cardiac health. This method not only shows promise in treating HF but also emphasizes the potential for long-term management strategies using inhalable therapies.

To facilitate the clinical translation of inhalation-based cardiovascular therapies such as dpCaP-MP or inhaled TP-10, the choice of physiologically relevant animal models is essential. While small animal models (e.g., pressure-overload or diabetic murine HF models) are indispensable for mechanistic studies, they exhibit fundamental anatomical and physiological differences from humans, particularly in lung architecture, airway branching, and cardiopulmonary interactions [40]. These limitations affect the predictability of aerosol deposition, drug translocation, and dosing. Conversely, large animal models, such as pigs, closely recapitulate human cardiopulmonary anatomy and disease progression, offering a more reliable platform for evaluating inhaled delivery systems. In our studies, consistent lung-to-heart nanoparticle translocation and robust therapeutic efficacy were demonstrated in both murine and porcine models, underscoring the translational viability of the LungToHeartNIM approach and related inhalation platforms [17, 29].

## Inhaled compounds for cardiovascular disease in clinical studies

Currently, no biologics have been tested for systemic targeting via inhalation. However, several compounds are already going through the market approval path. Recently, the results of a Phase 4 trial INHALE-3 study comparing inhalable insulin to current standard treatments in type 1 diabetes demonstrated that inhaled insulin (technosphere insulin, Afrezza, administered as a dry powder via a single puff) can be as effective as usual care while offering potential advantages in post-meal glucose control and patient preference (NCT05904743). Previous trials with the same insulin powder have demonstrated a potential bronchoconstriction in chronic lung disease, so that it currently contraindicated in those patients [41].

In a recent phase II open-label, multicenter study [42], the administration of flecainide (flecainide acetate solution), an old antiarrhythmic drug, via oral inhalation (two separate 3.5 min inhalations via an aerosol separated by a 1-min break) was shown to be safe and to yield plasma concentrations of flecainide sufficient to restore sinus rhythm in patients with recent-onset atrial fibrillation, leading to a Phase III study currently ongoing (RESTORE-1 trial, NCT05039359).

AV-101, a dry powder inhaled formulation of imatinib developed for pulmonary arterial hypertension, was successfully tested for safety and systemic exposure in healthy volunteers [43], while did not meet the primary endpoint of pulmonary vascular resistance reduction over 24 weeks in the IMPAHCT study (NCT05036135).

Finally worth mentioning is a route closely related to inhalation, the nasal administration. A recent, randomized study comparing a novel intranasal liquid formulation of the loop-diuretic bumetanide to oral and intravenous formulation showed a bioequivalence between oral and nasal formulations for key pharmacokinetic parameters. However, the faster absorption with the nasal route combined with similar efficacy and fewer side effects makes it a promising alternative for HF patients [44].

## Challenges and efficacy of inhaled therapies

Despite the potential benefits, the clinical development of inhaled therapies in the cardiovascular space remains challenging [11]. One of the key challenges in preclinical translation is achieving consistent pulmonary deposition and systemic bioavailability while minimizing clearance by alveolar macrophages and recruited neutrophils [45]. Inhaled nanoparticles must be engineered with specific physicochemical properties, including a size below 200 nm, neutral or slightly negative surface charge, and hydrophilic coatings (e.g., polyethylene glycol, PEG) to reduce recognition and uptake by immune cells, prolonging pulmonary residence and enhancing translocation into the systemic circulation. Additionally, biomimetic surface modifications (e.g., cell membrane cloaking) can create a “stealth” effect, minimizing immune activation and bystander effects in lung tissue [46].

Although biologics such as peptides and miRNAs are inherently low in immunogenicity, long-term inhalation therapy necessitates careful monitoring for immune activation. Our preclinical models did not show evidence of inflammatory or immunogenic responses following repeated dosing. Future trials will include specific immunotoxicity endpoints to address this concern.

Another critical consideration is ensuring drug stability and controlled release. Encapsulation strategies must balance protection of the therapeutic cargo with release kinetics suited to the target tissue. Technologies such as the NIM platform, where nanoparticles are embedded into inhalable microparticles, have demonstrated improved aerosol performance, protection of fragile biologics, and enhanced translocation to systemic targets [32].

To improve clinical translation, ongoing innovations focus on optimizing nanoparticle design, such as tuning size, shape, and surface chemistry for alveolar absorption and tissue targeting. PEGylation and ligand attachment can enhance circulation time and organ specificity, while pH-sensitive or enzyme-responsive materials allow for triggered release following cellular uptake. In parallel, efforts toward scalable GMP-compliant production, predictive large-animal models, and early-phase clinical trials are critical to validate both efficacy and pulmonary safety [47].

Patient-specific factors must also be addressed. Lung congestion, a common feature in HF, can potentially impact the safety and efficacy of inhaled medications by affecting drug deposition and absorption in the lungs and ensuring a consistent dosing of patients has been shown to be a challenge. Lung safety is crucial in patients with a chronic disease affecting the heart, given the frequent congestion and therefore susceptibility to side effects. To minimize bystander effects in the lung, inhaled biologics should employ surface-modified nanoparticles or pH-sensitive carriers that ensure drug release occurs only after cellular uptake. We previously demonstrated that inhaled CaP -based formulations are well tolerated in both healthy and diseased animals, including mice with diabetic cardiomyopathy and pigs with tachypacing-induced heart failure. No signs of lung toxicity, inflammation, or structural damage were observed, supporting their safety for systemic delivery via the pulmonary route [17, 29].

Inhalation-based drug delivery in critically ill, mechanically ventilated patients presents both challenges and opportunities. Factors such as ventilator settings, humidification, airway resistance, and device placement can significantly affect aerosol deposition and lung distribution. However, aerosolized formulations using vibrating mesh nebulizers or dry powder inhalers integrated into closed circuits have shown promise in optimizing pulmonary drug delivery under these conditions [48].

Finally, the convenience and ease of use of the inhalation device are pivotal to ensure patient adherence. In this regard, a lot has been done in the field of clinical inhalation science, with the launch of smart inhalers, including an app for home-monitoring of proper device use [49]. Therefore, while inhaled drugs hold promise for HF management, careful consideration of their long-term safety profiles is essential.

## Conclusions and future directions

The novel field of *cardiovascular Inhalation* is rapidly developing, with a substantial amount of research investigating different nanocarriers and disease targets being conducted by different labs worldwide, but ongoing research continues to refine these approaches. Innovations such as peptide-loaded nanoparticles demonstrate significant potential for enhancing drug delivery to the heart while minimizing systemic side effects. As studies progress, understanding the balance between efficacy and safety will be crucial in establishing inhaled drugs as a standard treatment modality in HF management. The next phase of clinical translation will require GMP-scale manufacturing, validated large-animal toxicology models, and early-phase clinical studies to confirm human safety and delivery efficacy. Our team is preparing a phase I trial for the LungToHeartNIM platform to explore these aspects in a controlled clinical setting.

In summary, inhaled drugs represent a novel frontier in HF treatment with the potential for targeted delivery and improved patient outcomes. However, further research is needed to fully understand their clinical effectiveness and long-term implications on lung health.

## Data Availability

No datasets were generated or analysed during the current study.
